# Refined THI Models for Evaluating the Effects of Heat Stress on Egg Production in Thai Native and Black-Boned Chickens

**DOI:** 10.3390/ani16131966

**Published:** 2026-06-25

**Authors:** Doungnapa Promket, Khanitta Pengmeesri, Vibuntita Chankitisakul, Wuttigrai Boonkum

**Affiliations:** 1 Branch of Animal Science, Department of Agricultural Technology, Faculty of Technology, Mahasarakham University, Mahasarakham 44150, Thailand; napakran@hotmail.com (D.P.); khanitta.c@msu.ac.th (K.P.); 2 Applied Animal and Aquatic Sciences Research Unit, Faculty of Technology, Mahasarakham University, Mahasarakham 44150, Thailand; 3 Department of Animal Science, Faculty of Agriculture, Khon Kaen University, Khon Kaen 40002, Thailand; vibuch@kku.ac.th; 4 Network Center for Animal Breeding and Omics Research, Khon Kaen University, Khon Kaen 40002, Thailand

**Keywords:** temperature–humidity index, test-day egg, thermal tolerance, management, indigenous breed

## Abstract

Heat stress is a major constraint on poultry productivity in tropical environments, where persistent high temperature and humidity intensify its negative effects on production traits. In this study, we quantified the relationship between thermal load and monthly egg production in black-boned and Thai native chickens and developed a refined temperature–humidity index intended to improve the assessment of heat stress under tropical conditions. A large dataset comprising 136,816 monthly egg production records from 11,530 birds was analyzed using regression models and seven THI equations. The results confirmed that heat stress significantly reduces monthly egg production, while conventional indices showed only moderate explanatory power. In contrast, the refined index consistently improved model performance, providing modest improvements in model fit compared with the original formulation. Notably, genotype-specific responses were identified, with Thai native chickens exhibiting greater tolerance to elevated thermal conditions. Distinct heat stress thresholds were also established, with values of 72 for black-boned and 74 for Thai native chickens. These findings highlight the environmentally sensitive nature of monthly egg production traits and demonstrate that targeted refinement of thermal indices enhances the detection of heat stress effects. This study provides a practical framework for integrating environmental indicators into management and breeding strategies aimed at improving thermal resilience in poultry systems.

## Simple Summary

Hot and humid weather can reduce egg production in chickens, especially in tropical areas where both temperature and humidity are high. In this study, we tested different ways to measure heat stress and refined existing THI approaches to better characterize heat stress effects in chickens. This refined approach may help identify the onset of heat-related reductions in monthly egg production more consistently. Our findings give farmers simple and practical tools to manage heat stress, such as adjusting housing, improving ventilation, and changing feeding practices at the right time. We also found that some chickens cope better with heat than others, which can help guide future breeding. Overall, this work supports the development of more heat-tolerant chickens and helps farmers maintain production under changing climate conditions.

## 1. Introduction

Accurate quantification of heat stress is fundamental to improving resilience and sustaining productivity in poultry systems under climate change [1,2]. However, existing temperature–humidity index (THI) models, largely developed under temperate conditions, may not adequately represent the nonlinear and genotype-dependent responses observed in native chicken populations raised in tropical environments. Egg production in poultry is a complex trait influenced by both genetic and environmental factors [3,4]. Among environmental stressors, heat stress is one of the most critical constraints affecting productivity, particularly in tropical and subtropical regions [5,6]. Elevated ambient temperature combined with high relative humidity impairs thermoregulation, leading to reduced feed intake, endocrine disruption, and ultimately declines in egg production and quality [7,8,9]. As climate change intensifies, understanding and mitigating heat stress effects has become essential for sustainable poultry production.

THI is widely used as an integrated indicator of heat stress in livestock, including poultry [10,11,12]. By incorporating both temperature and humidity, THI provides a more comprehensive estimate of thermal load than ambient temperature alone [11,13]. Increasing THI has been consistently associated with reductions in egg production, egg weight, and feed efficiency [11,14,15,16]. However, most existing THI formulations were originally developed for temperate conditions or for other livestock species and are typically applied assuming linear responses. This may limit their biological relevance for poultry, particularly for indigenous breeds raised under tropical environments.

Emerging evidence indicates that the relationship between THI and egg production is inherently nonlinear, with threshold effects and accelerated performance decline beyond critical physiological limits [17,18,19]. Under moderate thermal conditions, egg production may remain relatively stable but declines sharply once THI exceeds breed-specific tolerance thresholds [14,20]. Such nonlinear dynamics reflect cumulative stress effects and physiological tipping points [21,22]. Importantly, these thresholds are likely to vary across genetic backgrounds and production systems, suggesting that a single generalized THI may not adequately capture heat stress responses across diverse poultry populations.

Genetic background plays a key role in determining resilience to heat stress. Indigenous and locally adapted chicken breeds are generally more tolerant to harsh environmental conditions than highly selected commercial lines, although they often exhibit lower production potential [23,24,25]. Thai native chickens have shown relatively stable performance under tropical conditions [16,26], while black-boned chickens may differ in their physiological response to heat stress. Despite their importance, comparative studies evaluating nonlinear THI responses between these genetic groups remain limited. In addition, most previous studies have relied on cumulative or period-based egg production records, which may mask short-term responses to environmental fluctuations [14]. In contrast, test-day records provide a more precise representation of production dynamics and enable the detection of acute responses to changing thermal conditions [16,26].

These limitations highlight the need to develop a refined THI framework that better reflects the nonlinear, breed-specific responses of native chickens under tropical and subtropical conditions. Such an approach is essential for improving the accuracy of heat stress assessment, identifying biologically meaningful thresholds, and supporting more effective breeding and management strategies. Therefore, this study aimed to (1) characterize monthly egg production responses across varying THI levels, (2) develop a refined THI model tailored to native chicken populations, and (3) compare heat stress responses between the two breeds. The findings are expected to contribute to understanding heat stress resilience and contribute to the development of sustainable poultry production systems under changing climatic conditions.

## 2. Materials and Methods

All experimental procedures were approved by the Institutional Animal Care and Use Committee (IACUC) of Khon Kaen University (KKU) under the Ethical Guidelines for Animal Experimentation of the National Research Council of Thailand (Approval No. IACUC-KKU-108/68).

### 2.1. Animals and Management

**Figure 1:**
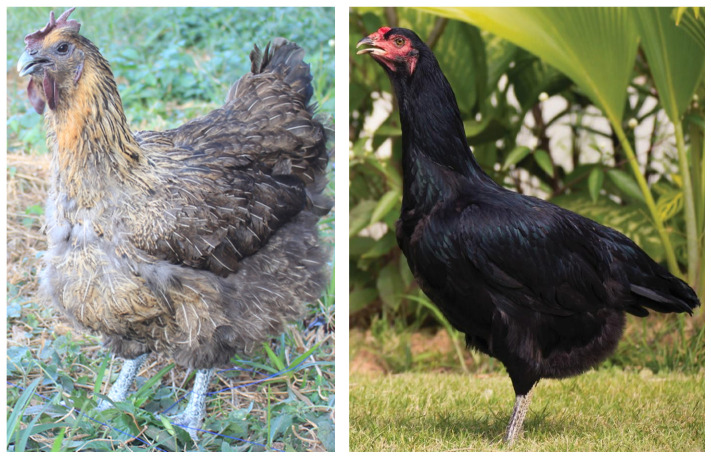
Representative photographs of the black-boned chicken (**left**) and Thai native chicken (**right**) used in this study.

The dataset comprised 43,220 and 93,596 monthly egg production records from 3680 black-boned chickens (Hmong) and 7850 Thai native chickens (Pradu Hang Dum), respectively. All birds were maintained at the experimental farm of the Network Center for Animal Breeding and Omics Research, Faculty of Agriculture, Khon Kaen University, Khon Kaen, Thailand. The two breeds shared similar morphological characteristics, including black feathers, beak, and shanks; however, they differed in skin pigmentation, with Thai native chickens exhibiting yellow skin and Hmong black-boned chickens showing black skin (Figure 1). The study was conducted from March 2020 to April 2026. At hatch, chicks were individually identified using leg bands and reared under standard brooding conditions for 4 weeks. Thereafter, birds were fitted with wing tags for permanent identification throughout the experimental period. Birds were vaccinated against infectious bronchitis, Newcastle disease, fowl pox, and fowl cholera following the standard vaccination program of the Department of Livestock Development, Thailand. Chickens were housed in an open-sided system under natural environmental conditions, with an average photoperiod of approximately 12 h of light per day. During the brooding phase (0–4 weeks), birds were fed a commercial starter diet containing 19% crude protein and 2900 kcal metabolizable energy (ME)/kg. From 4 to 20 weeks of age, a grower diet containing 15% crude protein and 2900 kcal ME/kg was provided. Feed and fresh water were available ad libitum during this period. At 20 weeks of age, hens were transferred to individual battery cages (20 cm × 45 cm × 40 cm; width × length × height) in accordance with animal welfare guidelines established by the Department of Livestock Development, Thailand. Egg production was recorded monthly from the onset of lay until 365 days in production. During the laying period, hens were provided a layer diet at 110 g/bird/day, containing 17% crude protein and 2750 kcal ME/kg, with continuous access to fresh water.

### 2.2. Environmental Data

**Figure 2:**
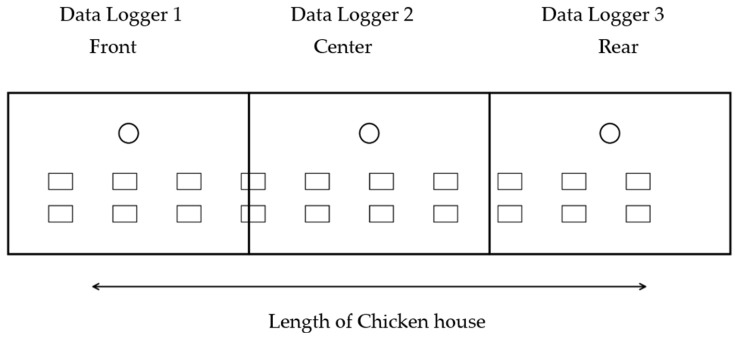
Schematic illustration of environmental sensor placement within the open-sided housing system. Three data loggers (ELITECH GSP-6, Elitech Technology Inc., USA) were positioned at bird level in the front, center, and rear sections of the house to record ambient temperature and relative humidity and capture spatial variation in microclimatic conditions during the study period.

To evaluate the effects of heat stress on monthly egg production trait, ambient temperature (T, °C) and relative humidity (RH, %) were recorded daily using three data loggers (ELITECH GSP-6, Elitech Technology Inc., Milpitas, CA, USA). The devices were installed at three representative locations within the housing system: at bird level in the central area, at the front, and at the rear of the house, to capture spatial variation in microclimatic conditions (Figure 2). Based on these measurements, THI values were calculated using established formulations. To account for the biological lag between environmental exposure and monthly egg production, seven THI functions were evaluated. For each formulation, THI was calculated as the average of the 30 days preceding each monthly egg production record. This period was selected to reflect the cumulative influence of environmental conditions on ovarian follicular development and egg formation in laying hens. Although the rapid growth phase of preovulatory follicles occurs over approximately 7–10 days, follicle recruitment, follicular hierarchy development, yolk deposition, and endocrine processes regulating ovulation extend over several weeks. Therefore, the use of a 30-day average THI was intended to capture the biologically relevant cumulative effects of thermal stress on reproductive performance rather than short-term environmental fluctuations [27,28].

### 2.3. Selecting the Optimal THI Function

All statistical analyses were performed using SAS software (version 9.0; SAS Institute Inc., Cary, NC, USA). Descriptive statistics were first computed to summarize the distribution of monthly egg production and environmental variables. Prior to analysis, data were screened for outliers and assessed for normality. Outliers were identified using PROC UNIVARIATE in SAS, together with boxplots and studentized residuals. Observations exceeding ±3 standard deviations from the mean were examined individually and removed only when deemed biologically implausible or attributable to recording errors. Normality was evaluated using the Shapiro–Wilk test and further verified by visual examination of residual histograms and normal quantile–quantile (Q–Q) plots. Differences between black-boned and Thai native chickens in monthly egg production, annual egg production, and age at first egg were analyzed using a general linear model (PROC GLM), with breed included as a fixed effect. Least-squares means were compared, and statistical significance was declared at *p* < 0.05.

Seven THI formulations previously developed for different livestock species were evaluated to identify the most suitable model for predicting monthly egg production. Each formulation differs in structure and underlying assumptions. THI1 and THI2 are based on linear combinations of average temperature and relative humidity, providing simple and widely used approaches for general heat stress assessment. However, their reliance on mean temperature may overlook diurnal fluctuations that are important for thermoregulation. THI3 incorporates interactions between temperature and humidity expressed in Fahrenheit and adjusted for thermal perception. Although commonly applied in human biometeorology, its direct applicability to livestock is limited and requires unit conversion from Celsius. THI4 considers only average temperature, excluding humidity, which reduces its suitability for humid tropical environments. In contrast, THI5 and THI6 incorporate diurnal variation through maximum and minimum temperatures, offering improved representation of thermal load. Nevertheless, these models do not account for humidity and rely on fixed weighting factors that may not be transferable across species or production systems. THI7 includes a nonlinear interaction between temperature and humidity and has been applied in tropical livestock studies, including poultry. To establish a baseline for model development, the most suitable existing THI formulation was identified and designated as the “original THI function.” Preliminary regression analyses were performed using PROC REG to evaluate the predictive performance of several published THI equations. Monthly egg production was modeled as a function of THI for each formulation. Model fit was assessed using the coefficient of determination (R^2^) and mean squared error (MSE). The equation with the highest R^2^ and lowest MSE was considered the most suitable for explaining variation in monthly egg production and was therefore selected as the baseline THI model for subsequent analyses. For each formulation, the following linear model was fitted:

$$Y_{i}=\mu +\beta_{1}\times {THI}_{i}+e_{i}$$

where

$Y_{i}\;$= monthly egg production record

μ = overall mean

$\beta_{1}$ = regression coefficient for THI

${THI}_{i}$ = temperature–humidity index

$e_{i}$ = residual error

The equations for the THI function are as follows [10,29,30,31,32,33,34]:

$$\mathrm{THI}1\;=\;\left(0.8\times T_{avg}\right)+\left[\left(\frac{{RH}_{avg}}{100}\right)\times \left(T_{avg}-14.4\right)\right]+46.4$$

$$\mathrm{THI}2=0.8\times T_{avg}+RH(T_{avg}-14.3)/100+46.3$$

$$\mathrm{THI}3=\left(1.8\times T_{avg}+32\right)-(0.55-0.0055\times {RH}_{avg})\times (1.8\times T_{avg}-26)$$

$$\mathrm{THI}4=T_{avg}+(0.36\times T_{avg})+41.2$$

$$\mathrm{THI}5=0.60\left(T_{max}\right)+0.40(T_{min})$$

$$\mathrm{THI}6=0.85\left(T_{max}\right)+0.15(T_{min})$$

$$\mathrm{THI}7=T_{avg}-\left(0.31-0.31\left(\frac{{RH}_{avg}}{100}\right)\right)\times \left(T_{avg}-14.4\right)$$

where ${{T_{min},T}_{max},T}_{avg}$ are the minimum, maximum, and average temperatures, respectively, in °C and ${RH}_{avg}$ is the average RH as a percentage.

### 2.4. Development of a New Temperature and Humidity Index

Following the identification of the best-performing THI formulation among the seven published equations, a series of refined THI models (THI_A to THI_J) was developed to improve the prediction of monthly egg production under tropical conditions. The coefficients for ambient temperature and relative humidity were systematically adjusted based on the original THI structure and evaluated using the training dataset (80% of the total records). Candidate equations were generated through iterative regression analyses using PROC GLM in SAS version 9.0, with monthly egg production as the response variable. A generalized form of the THI model was expressed as:

THI=a×T+b×RH

where T = ambient temperature (°C), RH = relative humidity (%), and a,  and b = regression coefficients to be estimated.

### 2.5. Model Fit and Validation

Because the objective of this study was to evaluate and compare the predictive performance of alternative THI formulations rather than estimate longitudinal effects of production factors, monthly egg production records were used as observational data for regression model development and validation. Model performance was evaluated using R^2^, MSE, AIC, and BIC. To assess the robustness of the refined THI model, THI thresholds ranging from 70 to 80 were examined to identify critical points associated with declines in monthly egg production. Internal validation was performed by randomly dividing the dataset into training and validation subsets using an 80:20 ratio. The training dataset was used to estimate model coefficients and determine the optimal THI threshold, whereas the validation dataset was used to evaluate predictive performance. In addition, year-based validation was conducted by fitting the model using records from earlier production years and evaluating its predictive ability using records from the most recent year.

## 3. Results

### 3.1. Data Structure and Descriptive Statistics

Data structure for estimation of variance components and genetic parameters in black-boned and Thai native chickens is presented in Table 1. Significant differences (*p* < 0.05) were observed between the two breeds for all production traits. Thai native chickens showed higher average test-day egg production (15.42 ± 3.0 eggs/month/bird) compared with black-boned chickens (14.08 ± 2.5 eggs/month/bird). Similarly, annual egg production was higher in Thai native chickens (185 ± 4.0 eggs/year/bird) than in black-boned chickens (169 ± 3.2 eggs/year/bird). In contrast, age at first egg differed between breeds, with Thai native chickens having a higher value (180 ± 29 days) compared with black-boned chickens (165 ± 24 days) (*p* < 0.05). The average environmental conditions across the study period were 28.1 ± 2.4 °C for air temperature and 77.5 ± 9.0% for relative humidity.

### 3.2. Appropriate Original THI for Egg Production Trait

The regression analysis between temperature–humidity index (THI) and monthly egg production traits is presented in Table 2. Across both breeds, THI1 consistently achieved the highest coefficient of determination (R^2^) and the lowest mean squared error (MSE), indicating superior predictive performance compared with the other formulations. In black-boned chickens, R^2^ values ranged from 0.480 to 0.488, whereas in Thai native chickens, they ranged from 0.445 to 0.457. Although differences among models were relatively small, the consistent superiority of THI1 across both breeds supported its selection as the baseline model for subsequent refinement and threshold analyses.

### 3.3. New THI Estimates

Comparisons between the original temperature–humidity index (THI1) and the newly developed THI functions (THI_A to THI_J) for monthly egg production traits are presented in Table 3. The validation analysis confirmed that the refined THI model maintained comparable performance between the training and validation datasets. Although the improvement over the original THI formulation was modest, the refined model showed consistent trends in both datasets, supporting its stability within the studied population. Model performance was evaluated using Akaike information criterion (AIC), Bayesian information criterion (BIC), coefficient of determination (R^2^), and mean squared error (MSE). The original THI1 showed an AIC of 4545.324, BIC of 3724.124, R^2^ of 0.457, and MSE of 8.389. All newly developed THI functions resulted in slight improvements in model fit, as indicated by lower AIC and BIC values and higher R^2^ compared with THI1. Among the tested models, THI_D provided the best fit, with the lowest AIC (4545.314) and BIC (3724.110), the highest R^2^ (0.465), and the lowest MSE (8.358). Similar performance was observed for THI_C and THI_E, which also showed improved model fit relative to THI1. Across the range of adjusted THI functions (THI_A to THI_J), AIC values varied minimally between 4545.314 and 4545.319, while BIC ranged from 3724.110 to 3724.119. The coefficient of determination (R^2^) ranged from 0.460 to 0.465, and MSE ranged from 8.358 to 8.363, indicating only marginal differences among the modified equations. Overall, the results indicate that the modified THI functions provided modest improvements in model performance compared with the original THI1, with THI_D yielding the most favorable fit based on all evaluated criteria.

### 3.4. Determination of Heat Stress Threshold Using the New THI

The onset of heat stress was identified using a threshold-search approach based on model-fit statistics rather than a predefined decline in monthly egg production, and the results are presented in Table 4. Candidate THI_D values ranging from 70 to 80 were evaluated sequentially. For each candidate value, a segmented heat-stress function was fitted in which the heat-stress effect was assumed to be absent below the threshold and to increase linearly above it. Model performance was evaluated using the sum of squared errors (SSE), Akaike information criterion (AIC), and Bayesian information criterion (BIC). The optimal THI_D was defined as the value that simultaneously minimized these criteria, indicating the point at which incorporating a heat-stress effect provided the best explanation of variation in monthly egg production. For black-boned chickens, the lowest SSE (533,024), AIC (1565), and BIC (1426) were observed at THI_D 72. In Thai native chickens, the corresponding minimum values occurred at THI_D 74 (SSE = 66,354; AIC = 1822; BIC = 1745). These values were therefore identified as the most likely thresholds at which egg production became increasingly sensitive to thermal stress. Although adjacent THI_D values produced similar results, they showed slightly poorer model fit, supporting the selection of THI_D 72 and THI_D 74 as the most appropriate thresholds for black-boned and Thai native chickens, respectively.

## 4. Discussion

This study provides a quantitative assessment of how thermal load, expressed through THI, influences monthly egg production in black-boned and Thai native chickens. The results demonstrate that environmental conditions, particularly the combined effects of temperature and relative humidity, significantly affect productive performance, consistent with previous studies identifying THI as a major indicator of heat stress in poultry [35,36]. Elevated THI has been associated with reduced monthly egg production, impaired reproductive performance, and altered metabolic activity in laying hens [5]. The observed breed differences, whereby Thai native chickens achieved higher egg production despite later sexual maturity, suggest distinct adaptive strategies under prolonged thermal challenge. Previous studies have reported that indigenous chicken breeds may exhibit greater adaptability to heat stress, although the physiological and molecular mechanisms underlying such responses were not directly evaluated in the present study [37,38]. These findings support the importance of considering genetic background when assessing productive performance under tropical conditions [15,16].

The moderate R^2^ values obtained across THI models indicate that thermal conditions explain a meaningful proportion of variation in monthly egg production but do not act independently. This agrees with previous studies demonstrating that heat stress responses are influenced by interactions among environmental, nutritional, physiological, genetic, and management factors [5,9,14]. Importantly, the present study extends previous work by evaluating indigenous chicken populations raised under tropical conditions, where chronic heat exposure represents a persistent production constraint. Similar studies have shown that native breeds display distinct reaction norms to THI while still experiencing measurable production declines as thermal load increases [15,16]. This supports the relevance of context-specific evaluation, particularly in climates where high temperature and humidity are chronic constraints [39].

Among the conventional indices, THI1 showed marginally superior predictive ability, confirming its utility as a baseline indicator of heat stress. However, the small differences among conventional THI formulations suggest that standard equations may not fully capture environmental and genetic variation under tropical production systems [40,41]. To address this limitation, modified THI equations were developed. Although improvements in model fit were modest, their consistency across evaluation criteria indicates that recalibration of temperature and humidity weighting can enhance predictive performance. Similar observations have been reported for reaction norm and locally calibrated THI models designed to better represent biological responses to heat stress [15]. The slightly improved performance of THI_D supports the development of context-specific models tailored to local climatic conditions and poultry genotypes. Nevertheless, because the improvement was relatively small, the refined model should be viewed as a population-specific calibration rather than a universal replacement for existing THI equations. Additional validation using independent populations, breeds, and environments will be required before broader application can be recommended.

A major outcome of this study was the identification of breed-specific heat stress thresholds. The heat stress thresholds of THI 72 for black-boned chickens and THI 74 for Thai native chickens indicate that Thai native chickens can tolerate slightly greater thermal loads before production declines occur. Similar genotype-dependent differences have been reported previously in poultry populations adapted to tropical environments [5,24]. Although the physiological mechanisms responsible for these differences were not investigated directly, previous studies have suggested that variation in thermoregulation, cellular stress responses, and metabolic adaptation may contribute to differences in heat tolerance among genotypes [16,39]. Therefore, the observed threshold differences likely reflect breed-specific adaptation to local environmental conditions.

The identification of breed-specific heat stress thresholds has important practical implications because it provides producers with quantitative benchmarks for environmental management. The threshold values identified in this study fall within the range previously reported for poultry [42,43] but offer greater precision by accounting for genotype-specific responses. As THI approaches critical levels, producers can proactively implement mitigation strategies to minimize adverse effects on egg production. Environmental interventions, including improved ventilation, shading, and evaporative cooling systems, may help reduce heat load and maintain thermal comfort. In addition, nutritional strategies can enhance resilience to heat stress. Dietary electrolyte supplementation, such as sodium bicarbonate, potassium chloride, and dietary canthaxanthin, has been shown to improve egg production in poultry exposed to high temperatures [2,9,44]. Likewise, antioxidants including vitamin C, vitamin E, selenium, and phytogenic compounds may alleviate oxidative stress and support physiological function under heat challenge [8,45]. Therefore, integrating THI-based environmental monitoring with targeted nutritional interventions may provide an effective approach for reducing production losses in tropical poultry systems. These findings are consistent with the growing adoption of precision livestock farming technologies that use environmental monitoring to improve management decisions and mitigate the adverse effects of heat stress on poultry performance [5,39]. Although an economic analysis was not performed, the observed decline in egg production beyond the identified thresholds suggests the potential for substantial cumulative production losses under prolonged heat stress. The implications of this work also extend to breeding programs. The observed differences in heat tolerance emphasize the importance of incorporating adaptive traits into selection objectives, particularly as climate change increases the frequency and severity of thermal stress. The refined THI model developed in this study provides a practical framework for linking environmental conditions with productive performance and may support future genetic evaluations that simultaneously consider productivity and resilience to heat stress.

An additional limitation of this study is that the refined THI model was based solely on ambient temperature and relative humidity. Although these variables form the basis of most heat-stress indices and explain a substantial proportion of the variation in egg production, they may not fully capture the thermal conditions experienced by birds. Factors such as wind speed, solar radiation, ventilation efficiency, and housing design can markedly influence heat exchange and thermal perception, particularly in open-sided poultry systems common in tropical regions. Therefore, future research should develop multi-variable heat-load models that incorporate climatic, environmental, and physiological indicators to improve the prediction of production responses under heat stress and enhance the applicability of thermal assessment tools across diverse production systems. Future studies incorporating temperature and relative humidity as separate predictors may benefit from advanced visualization approaches, such as contour plots and three-dimensional response surfaces, to further characterize nonlinear thermal responses and breed-specific heat tolerance under tropical production conditions. Furthermore, the applicability of the identified thresholds beyond the studied population requires validation under different production systems and climatic conditions. Future studies integrating genotype-by-environment analyses, physiological measurements, and genomic information may provide deeper insight into the biological basis of heat tolerance and support the development of more resilient poultry populations under tropical conditions.

## 5. Conclusions

This study demonstrated that heat stress significantly affects egg production in both black-boned and Thai native chickens under tropical conditions. Among the evaluated THI functions, the refined THI_D model provided the best overall fit, although its improvement over conventional THI models was relatively small. Breed-specific heat stress thresholds were identified at THI 72 for black-boned chickens and THI 74 for Thai native chickens, indicating greater thermal tolerance in the Thai native breed. These findings emphasize the importance of considering genetic background when evaluating heat stress responses and developing management strategies for poultry production in tropical environments. The refined THI_D model was developed using data from a single population under specific environmental conditions, which may limit its broader applicability. In addition, although the model provided the best fit among the evaluated formulations, the differences in predictive performance were relatively small. Therefore, the identified thresholds and model parameters should be validated across independent populations, production systems, and climatic regions before widespread implementation. In conclusion, breed-specific THI thresholds and locally calibrated thermal indices may improve the assessment of heat stress in indigenous chickens and provide practical tools for supporting climate-resilient management and genetic improvement programs. The refined THI_D model identified in this study represents a useful framework for future research, although further validation is required to confirm its robustness across diverse production environments.

## Figures and Tables

**Table 1:** Data structure for estimation of variance components and genetic parameters in black-boned and Thai native chickens.

Categories	Total	Chicken Breed	
		Black-Boned	Thai Native
Animals with records (n)	11,530	3680	7850
Animals with pedigrees (n)	28,590	8450	20,140
Number of records (n)	136,816	43,220	93,596
Average monthly egg production (eggs/month/bird ± SD)		14.08 ± 2.5 ^b^	15.42 ± 3.0 ^a^
Average annual egg production (eggs/year/bird ± SD)		169 ± 3.2 ^b^	185 ± 4.0 ^a^
Average age at first egg (days ± SD)		165 ± 24 ^b^	180 ± 29 ^a^
Average air temperature (°C ± SD)	28.1 ± 2.4	-	-
Average relative humidity (% ± SD)	77.5 ± 9.0	-	-

Different superscript letters (a, b) indicate significant differences within the same row (*p* < 0.05).

**Table 2:** Regression analysis of statistical parameters (R^2^ and MSE) between the temperature and humidity index (THI) and monthly egg production trait in black-boned and Thai native chickens.

Temperature–Humidity Index	Chicken Breed			
	Black-Boned Chicken		Thai Native Chicken	
	R^2^	MSE	R^2^	MSE
THI1	0.488	8.496	0.457	8.389
THI2	0.486	8.498	0.454	8.391
THI3	0.485	8.499	0.453	8.392
THI4	0.484	8.500	0.448	8.394
THI5	0.482	8.502	0.446	8.395
THI6	0.480	8.503	0.445	8.395
THI7	0.480	8.505	0.445	8.396

R^2^: coefficient of determination; MSE: mean squared error.

**Table 3:** Comparisons between original THI function and new THI functions with model fit statistics (AIC, BIC, R^2^, MSE).

Developing New THI	Egg Production Trait			
	AIC	BIC	R^2^	MSE
THI1 = $\left(0.8\times T_{avg}\right)+\left[\left(\frac{{RH}_{Avg}}{100}\right)\times \left(T_{avg}-14.4\right)\right]+46.4$	4545.324	3724.124	0.457	8.389
THI_A = $\left(0.8\times T_{avg}\right)+\left[\left(\frac{{RH}_{Avg}}{100}\right)\times \left(T_{avg}-20.1\right)\right]+45.1$	4545.317	3724.115	0.462	8.362
THI_B = $\left(0.8\times T_{avg}\right)+\left[\left(\frac{{RH}_{Avg}}{100}\right)\times \left(T_{avg}-20.2\right)\right]+45.1$	4545.317	3724.115	0.463	8.360
THI_C = $\left(0.8\times T_{avg}\right)+\left[\left(\frac{{RH}_{Avg}}{100}\right)\times \left(T_{avg}-20.3\right)\right]+45.1$	4545.315	3724.112	0.465	8.359
THI_D = $\left(0.8\times T_{avg}\right)+\left[\left(\frac{{RH}_{Avg}}{100}\right)\times \left(T_{avg}-20.4\right)\right]+45.1$	4545.314	3724.110	0.465	8.358
THI_E = $\left(0.8\times T_{avg}\right)+\left[\left(\frac{{RH}_{Avg}}{100}\right)\times \left(T_{avg}-20.5\right)\right]+45.1$	4545.316	3724.113	0.464	8.360
THI_F = $\left(0.8\times T_{avg}\right)+\left[\left(\frac{{RH}_{Avg}}{100}\right)\times \left(T_{avg}-20.6\right)\right]+45.1$	4545.316	3724.113	0.462	8.360
THI_G = $\left(0.8\times T_{avg}\right)+\left[\left(\frac{{RH}_{Avg}}{100}\right)\times \left(T_{avg}-20.7\right)\right]+45.1$	4545.317	3724.115	0.461	8.361
THI_H = $\left(0.8\times T_{avg}\right)+\left[\left(\frac{{RH}_{Avg}}{100}\right)\times \left(T_{avg}-20.8\right)\right]+45.1$	4545.317	3724.115	0.461	8.361
THI_I = $\left(0.8\times T_{avg}\right)+\left[\left(\frac{{RH}_{Avg}}{100}\right)\times \left(T_{avg}-20.9\right)\right]+45.1$	4545.319	3724.117	0.460	8.363
THI_J = $\left(0.8\times T_{avg}\right)+\left[\left(\frac{{RH}_{Avg}}{100}\right)\times \left(T_{avg}-21.0\right)\right]+45.1$	4545.319	3724.119	0.460	8.363

AIC: Akaike information criterion; BIC: Bayesian information criterion; R^2^: coefficient of determination; MSE: mean squared error.

**Table 4:** Testing of heat stress onset using newly developed THI (THI_D) in black-boned and Thai native chickens.

THI Threshold	THI_F In Black-Boned Chicken			THI_F in Thai Native Chicken		
	SSE	BIC	AIC	SSE	BIC	AIC
70	533,075	1435	1574	66,369	1762	1839
71	533,042	1430	1569	66,365	1758	1835
72	533,024	1426	1565	66,361	1753	1830
73	533,030	1429	1568	66,356	1749	1826
74	533,034	1432	1571	66,354	1745	1822
75	533,067	1436	1575	66,355	1748	1825
76	533,072	1439	1578	66,359	1750	1827
77	533,084	1442	1581	66,362	1756	1833
78	533,089	1445	1584	66,364	1760	1837
79	533,095	1449	1588	66,367	1768	1845
80	533,112	1457	1596	66,370	1775	1852

SSE: sum of squared errors, AIC: Akaike information criterion, BIC: Bayesian information criterion.

## Data Availability

The original contributions presented in the study are included in the article; further inquiries can be directed to the corresponding author.

## References

[1] Lallo C.H.O., Cohen J., Rankine D., Taylor M., Cambell J., Stephenson T. (2018). Characterizing heat stress on livestock using the temperature humidity index (THI)—Prospects for a warmer Caribbean. Reg. Environ. Change.

[2] Wasti S., Sah N., Mishra B. (2020). Impact of heat stress on poultry health and performances, and potential mitigation strategies. Animals.

[3] Nawaz A.H., Setthaya P., Feng C. (2024). Exploring evolutionary adaptations and genomic advancements to improve heat tolerance in chickens. Animals.

[4] Kadawarage R.W., Dunislawska A., Siwek M. (2024). Ecological footprint of poultry production and effect of environment on poultry genes. Phys. Sci. Rev..

[5] Idowu P.A., Chauke C., Mpofu T.J. (2026). Integrated stress physiology and mitigation strategies for heat stress in layer chickens—Review. Animals.

[6] Chaudhary A., Mishra B. (2024). Systemic effects of heat stress on poultry performances, transcriptomics, epigenetics and metabolomics. Worlds Poult. Sci. J..

[7] Lara L.J., Rostagno M.H. (2013). Impact of heat stress on poultry production. Animals.

[8] Mangan M., Siwek M. (2024). Strategies to combat heat stress in poultry production—A review. J. Anim. Physiol. Anim. Nutr..

[9] Olayiwola S.F., Adedokun S.A. (2025). Heat stress in poultry: The role of nutritional supplements in alleviating heat stress and enhancing gut health in poultry. Front. Vet. Sci..

[10] Tao X., Xin H. (2003). Acute synergistic effects of air temperature, humidity, and velocity on homeostasis of market-size broilers. Trans. ASAE.

[11] Kim D.-H., Lee Y.-K., Kim S.-H., Lee K.-W. (2021). The impact of temperature and humidity on the performance and physiology of laying hens. Animals.

[12] Du X., Carpentier L., Teng G., Liu M., Wang C., Norton T. (2020). Assessment of laying hens’ thermal comfort using sound technology. Sensors.

[13] El-Tarabany M.S. (2016). Impact of temperature–humidity index on egg-laying characteristics and related stress and immunity parameters of Japanese quails. Int. J. Biometeorol..

[14] Tesakul S., Mitsuwan W., Morita Y., Kitpipit W. (2025). Effects of heat stress on egg performance in laying hens under hot and humid conditions. Vet. World.

[15] Sungkhapreecha P., Chankitisakul V., Boonkum W. (2026). Genetic parameter estimates and associations between clutch length and hen-day egg production traits in Thai native chickens under heat stress. Animals.

[16] Promket D., Pengmeesri K., Chankitisakul V., Boonkum W. (2025). Comparative analysis of genetic parameters for test-day egg production in four Thai native synthetic chicken lines under heat stress. Animals.

[17] Cordeiro A.F., Nääs I.A., Garcia R.G., Valentim J.K. (2025). Machine learning-based decision trees to predict egg production performance in laying hens under heat stress conditions. Braz. J. Poult. Sci..

[18] Solis I.L., de Oliveira-Boreli F.P., de Sousa R.V., Martello L.S., Pereira D.F. (2024). Using thermal signature to evaluate heat stress levels in laying hens with a machine-learning-based classifier. Animals.

[19] Ha T., Kwon K., Hong S.W., Yeo U.H. (2026). Regression meta-model for predicting THI in poultry houses. Agriculture.

[20] Kim H.-R., Ryu C., Lee S.-D., Cho J.-H., Kang H. (2024). Effects of heat stress on the laying performance, egg quality, and physiological response of laying hens. Animals.

[21] Renaudeau D., Collin A., Yahav S., de Basilio V., Gourdine J.L., Collier R.J. (2012). Adaptation to hot climate and strategies to alleviate heat stress in livestock production. Animal.

[22] Asseng S., Spänkuch D., Hernández-Ochoa I.M., Laporta J. (2021). The upper temperature thresholds of life. Lancet Planet. Health.

[23] Padhi M.K. (2016). Importance of indigenous breeds of chicken for rural economy and their improvements for higher production performance. Scientifica.

[24] Fodor I., Spoelstra M., Calus M.P.L., Kamphuis C. (2023). A systematic review of genotype-by-climate interaction studies in cattle, pigs, and chicken. Front. Anim. Sci..

[25] Rovelli G., Ceccobelli S., Perini F., Demir E., Mastrangelo S., Conte G., Abeni F., Marletta D., Ciampolini R., Cassandro M. (2020). The genetics of phenotypic plasticity in livestock in the era of climate change: A review. Ital. J. Anim. Sci..

[26] Loengbudnark W., Chankitisakul V., Boonkum W. (2023). The genetic impact of heat stress on egg production in Thai native chickens (Pradu Hang Dum). PLoS ONE.

[27] Johnson A.L. (2015). Ovarian follicle selection and granulosa cell differentiation. Poult. Sci..

[28] Johnson A.L. (2014). The avian ovary and follicle development: Some comparative and practical insights. Turk. J. Vet. Anim. Sci..

[29] Mader T.L., Davis M.S., Brown-Brandl T. (2006). Environmental factors influencing heat stress in feedlot cattle. J. Anim. Sci..

[30] de Moraes S.R.P., Yanagi Júnior T., de Oliveira A.L.R., Yanagi S.N.M., Café M.B. Classification of the temperature and humidity index (THI), aptitude of the region, and conditions of comfort for broilers and layer hens in Brazil. Proceedings of the International Conference of Agricultural Engineering, XXXVII Brazilian Congress of Agricultural Engineering, International Livestock Environment Symposium (ILES VIII).

[31] National Oceanic and Atmospheric Administration (NOAA) (1976). Livestock Hot Weather Stress.

[32] Yousef M.K. (1985). Stress Physiology in Livestock. Volume I: Basic Principles.

[33] Zulovich J.M., DeShazer J.A. (1990). Estimating Egg Production Declines at High Environmental Temperatures and Humidities. *Paper—American Society of Agricultural Engineers*. https://www.cabidigitallibrary.org/doi/full/10.5555/19912449755.

[34] Marai I.F.M., Ayyat M.S., Abd El-Monem U.M. (2001). Growth performance and reproductive traits at first parity of New Zealand White female rabbits as affected by heat stress and its alleviation under Egyptian conditions. Trop. Anim. Health Prod..

[35] Perini F., Cendron F., Rovelli G., Castellini C. (2021). Emerging genetic tools to investigate molecular pathways related to heat stress in chickens: A review. Animals.

[36] Jongbo A.O., de Borba L.P., Pereira R.M.M., Bello Q.O., Gregoratto L.L., de Souza D.P., Adeyeye O.A., Vieira F.M.C. (2026). Heat stress in livestock under tropical climates: Impacts and mitigation strategies. Trop. Anim. Health Prod..

[37] Rachman M.P., Bamidele O., Dessie T., Smith J., Hanotte O., Gheyas A.A. (2024). Genomic analysis of Nigerian indigenous chickens reveals their genetic diversity and adaptation to heat stress. Sci. Rep..

[38] Hossain M.M., Ahn J., Choi S.-Y., Hur S.-P., Lim D., Shin D., Lee S., Park J.-E. (2026). Thermal stress responses and heat stress resilience genes in chickens revealed through genomic and transcriptomic insights. J. Anim. Sci. Biotechnol..

[39] Al-Khalaifah H.S., Al-Nasser A.Y., Suleiman M.K., Shahid S.A. (2026). Effect of rising temperatures on overall health, productive and reproductive performance of chickens—A review. Fostering Arid Lands Agriculture in the Face of Climate Change.

[40] Peng H., Wang Y., Zhang Z., Qin W., Li B., Zheng W., Yin P., Zhu H. (2024). Effects of low-pressure systems on temperature, humidity, egg production, and feed utilization efficiency in large-scale poultry houses during summer. Animals.

[41] Pranta A.M., Islam S.M.A., Khan R.I. (2025). Development of a sensor-integrated AI automation model for decision-based heat stress management in layer chickens under subtropical climate conditions. Smart Agric. Technol..

[42] Eletu T.A., Anjola I.A., Fasasi L.O., Fadipe D.P., Afuye A.T., Fasanya F.O., Oke O.E. (2026). Heat stress in quail: Impacts on health and productivity, and mitigation strategies. Anim. Res. One Health.

[43] Wankar A.K., Bhangale G.N., Rindhe S.N., Kumawat B.L., Shafi T.A. (2024). Heat stress in beef cattle: Climate change and the global scenario—A review. Ann. Anim. Sci..

[44] Zhou X., Song Y., Chen J., Chen X., Guan L., Wang Y., Xiao M., Liu W., An L. (2025). Effects of dietary canthaxanthin on egg production, serum parameters, and intestinal health in indigenous chickens under heat stress. Front. Vet. Sci..

[45] Shakeri M., Oskoueian E., Le H.H., Shakeri M. (2020). Strategies to combat heat stress in broiler chickens: Unveiling the roles of selenium, vitamin e and vitamin c. Vet. Sci..

